# Calcium, Potassium, Sodium, and Magnesium Concentrations in the Placenta, Umbilical Cord, and Fetal Membrane from Women with Multiple Pregnancies

**DOI:** 10.3390/life13010153

**Published:** 2023-01-05

**Authors:** Konrad Grzeszczak, Patrycja Kapczuk, Patrycja Kupnicka, Elżbieta Cecerska-Heryć, Sebastian Kwiatkowski, Dariusz Chlubek, Danuta Kosik-Bogacka

**Affiliations:** 1 Department of Biology and Medical Parasitology, Pomeranian Medical University in Szczecin, Powstańców Wielkopolskich 72, 70-111 Szczecin, Poland; 2 Department of Biochemistry and Medical Chemistry, Pomeranian Medical University in Szczecin, Powstańców Wielkopolskich 72, 70-111 Szczecin, Poland; 3 Department of Laboratory Medicine, Pomeranian Medical University in Szczecin, Powstańców Wielkopolskich 72, 70-111 Szczecin, Poland; 4 Department of Obstetrics and Gynecology, Pomeranian Medical University in Szczecin, Powstańców Wielkopolskich 72, 70-111 Szczecin, Poland; 5 Independent Laboratory of Pharmaceutical Botany, Pomeranian Medical University in Szczecin, Powstańców Wielkopolskich 72, 70-111 Szczecin, Poland

**Keywords:** calcium, potassium, sodium, magnesium, pregnancy complications, preeclampsia, pregnancy

## Abstract

Calcium (Ca), potassium (K), sodium (Na), and magnesium (Mg) are the elements responsible for the fundamental metabolic and biochemical processes in the cells of the body. The demand for these elements increases significantly during pregnancy, where an adequate supply protects women from the hypertension common in pre-eclampsia and preterm labor. This study aimed to evaluate the association between macro-elements (Ca, Mg, Na, and K) in the placenta, fetal membrane, and umbilical cord and the morphometric parameters of newborns from multiple pregnancies. The study involved 57 pregnant European women with healthy uncomplicated twin pregnancies (*n* = 52) and triple pregnancies (*n* = 5); 40 pairs of dichorionic diamniotic twins, 11 pairs of monochorionic diamniotic twins, 1 pair of monochorionic monoamniotic twins, 3 trichorionic triamniotic triplets, and 2 dichorionic triamniotic triplets. Placentas (*n* = 107), umbilical cords (*n* = 114), and fetal membranes (*n* = 112) were collected immediately following delivery, and then weighed and measured. The levels of Ca, K, Na, and Mg were determined using inductively coupled plasma atomic emission spectroscopy (ICP-OES) in a Thermo Scientific ICAP 7400 Duo (Waltham, MA, USA). The respective mean concentrations of Ca, K, Na, and Mg (mg/kg^−1^ dry mass) were: 2466, 8873, 9323, and 436 in the placenta; 957, 6173, 26,757, and 326 in the umbilical cord, and 1252, 7460, 13,562, and 370 in the fetal membrane. In the studied materials from northwestern Poland, we found strong positive correlations between Ca and Mg concentrations in both the umbilical cord (r = 0.81, *p* = 0.00) and the fetal membrane (r = 0.73, *p* = 0.00); between K and Mg concentrations in the umbilical cord (r = 0.73, *p* = 0.00); between Ca and K concentrations in the fetal membrane (r = 0.73, *p* = 0.00), and we found moderately positive correlations between placental Ca concentration and placental weight (ρ = 0.42, *p* = 0.00) and between umbilical cord Mg concentrations and the length of the pregnancy (ρ = 0.42, *p* = 0.00). Negative correlations were found between Na and Ca concentrations in the fetal membrane (r = −0.40, *p* = 0.00) and Na concentrations in the fetal membrane and Mg concentrations in the placenta (r = −0.16, *p* = 0.02). Negative correlations were confirmed between the length of pregnancy and head circumference (ρ = −0.42; p = 0.00), infant weight (ρ = −0.42; *p* = 0.00), infant length (ρ = −0.49; *p* = 0.00), shoulder width (ρ = −0.49; *p* = 0.00); and between the infant weight and head circumference (ρ = −0.62; *p* = 0.00), weight before delivery (ρ = −0.36; *p* = 0.00), infant length (ρ = −0.45; *p* = 0.00), shoulder width (ρ = −0.63; *p* = 0.00), and weight gain during pregnancy (ρ = −0.31; *p* = 0.01). We found statistically significant correlations between cigarette smoking before pregnancy and the women’s weight before delivery (ρ = 0.32, *p* = 0.00), and a negative correlation between the women’s ages and infant head circumference (ρ = −0.20, *p* = 0.02). This is probably the first study to evaluate Ca, Na, K, and Mg concentrations in the afterbirth tissues of multiple pregnancies. It adds to the knowledge of elemental concentrations in multiple pregnancies and their possible effects on fetal morphometric parameters.

## 1. Introduction

Essential and non-essential elements ensure normal human development, reproduction, and health throughout life [1]. Among them, calcium (Ca), potassium (K), sodium (Na), and magnesium (Mg) are responsible for metabolic and biochemical processes in the cells of the body and the maintenance of proper pH, osmotic pressure, nerve conduction, muscle contraction, and heart rhythm [2,3].

During pregnancy, the mineral metabolism of the mother must adapt to the increased demand for elements created by the fetus and placenta. As a key micronutrient, Ca plays different roles during fetus growth and development [4]. According to current recommendations, the average requirements (AR) for Ca for pregnant women aged between 18 and 24 and those above 25 are, respectively, 860 and 750 mg/day, and the population reference intake (PRI) is 1000 and 950 mg/day, respectively. For pregnant and lactating women, the Tolerable Upper Intake Level (UL) should be 2500 mg of Ca from both the diet and supplementation (EFSA) [5]. During pregnancy, there is a significant increase in intestinal Ca^2+^ absorption, which is mediated by the active metabolite of vitamin D_3_ 1,25-dihydroxycholecalciferol (1,25-(OH)_2_D_3_, calcitriol), parathyroid hormone (PTH), and calcitonin [6,7]. In the second trimester, Ca absorption is 57% higher, increasing to 72% higher (250–350 mg) in the third trimester—the period of intense fetal bone growth [4]. The fetal Ca concentration increases exponentially during pregnancy [8]. Calcium is delivered to the fetus via three routes: transport to the placental syncytiotrophoblast from the maternal blood via the epithelial calcium channel; Ca transfer to the fetal-facing basal membrane by Ca-binding proteins, including calbindin-D9K; and via the cell membrane calcium ATPase, which transports Ca across the fetal-facing basal membrane [9]. Jankowska et al. [10] have shown that the majority of women in Poland do not satisfy the need for the most necessary dietary elements, such as Ca. Polish recommendations in 2011 for Ca stated 1200 mg per day; however, these are recommendations for women with a single pregnancy [11]. Inadequate Ca intake during pregnancy leads to decreased bone density in both the mother and child. It can also lead to pregnancy-induced hypertension, a risk factor for preterm delivery, low birth weight, and prenatal and maternal deaths [12].

Potassium (K) is an essential element for a normal pregnancy, with a recommended adequate intake (AI) for pregnant women above 18 of 3500 mg/day (EFSA) [5]. It is mainly absorbed in the small intestine and distal colon with ATPase [13]. There is no proven effect of maternal hypokalemia on fetal parameters in humans, due to the efficient compensation of K deficiency by the placenta. However, animal model studies have shown that K deficiency impairs fetal growth [14]. Hyperkalemia, on the other hand, can cause the development of gestational diabetes mellitus (GDM) and severe pre-eclampsia [15]. Gestational diabetes mellitus impairs normal fetal growth, and pre-eclampsia limits fetal growth and can result in preterm labor [16].

Sodium is the most important electrolyte in extracellular fluids [17]. An intake of 2 g/day is considered a safe and adequate level, consistent with the reference value set for adults (EFSA) [5]. It is absorbed from the intestines along with glucose and amino acids by Na^+^/H^+^ exchange and then is transported to enterocytes [18]. To the fetus, Na is transferred via the syncytiotrophoblast Na^+^/K^+^-ATPase of the placenta [19]. Maternal hyponatremia has been found to affect the occurrence of low birth weight, spontaneous abortion, or impaired fetal growth [20].

Magnesium (Mg), a Ca antagonist [21], is the second most important intracellular cation after potassium [22]. The recommended intake of Mg for pregnant women over 18 is 300 mg/day, while the maximum UL should not exceed 250 mg/day (EFSA) [5]. The Polish recommendations from 2011 are 200–1000 Mg per day, but only concern women with a single pregnancy [11]. Magnesium is absorbed in the jejunum and ileum via passive transport related to the electrochemical gradient and diffusion via the transient receptor potential melastatin type 6 (TRPM6) ion channel [23]. High Mg concentrations in the placenta may reduce Ca transport to the fetus [24]. Magnesium influences potassium channels and can regulate its placental transport. Despite the use of supplementation, many women have inadequate Mg levels [10]. Magnesium deficiency during pregnancy can result in hypertension, pre-eclampsia, painful muscle contraction, and migraines [25]. In addition, Mg deficiency increases the risk of premature birth, gestational diabetes, and fetal growth disorders [26]. Increased Mg intake during pregnancy is associated with increased birth weight [27]. Magnesium supplementation is associated with lower incidences of both preterm births and low birth weight [28]. However, hypermagnesemia has toxic effects on pregnancy, leading to diminished deep tendon reflexes, apnoea, and electromechanical dissociation [29].

The number of multiple pregnancies has increased significantly in recent years. This is related to the use of assisted reproductive techniques—induced ovulation and in vitro fertilization. The average duration of a single pregnancy is 39 weeks, while multiple pregnancies last, respectively, for twins 36 weeks, for triplets 32 weeks, and for quadruplets 30 weeks [30,31]. The variation in multiple birth rates across Europe in 2010 was classified based on four groups of countries defined by multiple birth rates. The lowest group (less than 15 twin births per 1000 women) included Central and Eastern European countries (Romania, Latvia, Lithuania, Poland, and Slovakia) and some Scandinavian countries (Iceland and Sweden). The second group (15.0 to 16.9 per 1000) included Estonia, Portugal, Finland, the United Kingdom, Italy, Norway, and Ireland, and the third group (17.0 to 18.9 per 1000) included Austria, France, the Netherlands, Luxembourg, Switzerland, Slovenia, and Germany. The highest group (≥19 per 1000) comprised Belgium, Malta, Spain, Denmark, the Czech Republic, and Cyprus [32].

Multiple pregnancies are always higher-risk pregnancies. Women with multiple pregnancies have a higher rate of pregnancy complications, fetal malformations, and perinatal morbidity and mortality than women with single pregnancies [30]. Multiple pregnancies can result in preterm labor, miscarriage, hypotrophy of both or one fetus, intrauterine fetal demise, or fetal atrophy syndrome [33]. Preeclampsia is at least 2–3 times more common and is generally more severe in twin pregnancies than in singleton pregnancies [34].

As micronutrient deficiencies can lead to serious long-term health consequences for both mother and baby, it is very important to identify women at risk of these complications as early as possible. Many studies address the role of Ca during pregnancy, but there are fewer data on Ca supplementation in patients with multiple pregnancies. Also, there are few available studies on Mg, Na, and K, and the results are inconclusive. That is why this study aimed at evaluating the concentration of elements that play an important role during pregnancy (Ca, Mg, Na, and K) in the placenta, fetal membrane, and umbilical cord collected from women with multiple pregnancies from northwest Poland, as well as evaluating associations between the studied elements and the morphometric parameters of newborns from multiple pregnancies. The analysis of elemental concentrations in the afterbirth can contribute to a better understanding of pregnancy disorders and the possibility of using such data as a means of predicting the course of a pregnancy.

## 2. Materials and Methods

### 2.1. Ethics Statement

This study was conducted from 2015 to 2021. The research was carried out with the approval of the Biometric Committee of the Pomeranian Medical University in Szczecin (KB-0012/76/14 from 13 October 2014). The study was conducted in accordance with the Declaration of Helsinki. Patients gave written consent to participate and were informed that they could withdraw their consent at any stage of the study.

### 2.2. Study Population

The study involved pregnant women from north-west Poland (*n* = 57), comprising healthy un-complicated twin pregnancies (*n* = 52) and triple pregnancies (*n* = 5),and newborns (*n* = 114), delivered at the Obstetrics and Gynecology Clinic of the independent Public Clinical Hospital No. 2 of the Pomeranian Medical University of Szczecin, Szczecin, The West Pomeranian Voivodeship, Poland. The West Pomeranian area is moderate in terms of environmental conditions. This region is characterized by developed agriculture and the food production industry. Other important industries mainly include wood processing, chemicals, metallurgy, shipbuilding, and electricity production.

All of these births were resolved by cesarean section. The inclusion criteria for the study were multiple pregnancies and newborns without perinatal illness, and the group was randomly collected. All the women were without risk factors that could affect neonatal parameters. Infants with anemia and chromosomal abnormalities and/or birth defects were also excluded from the study.

The study comprised 40 pairs of dichorionic diamniotic twins, 11 pairs of monochorionic diamniotic twins, 1 pair of monochorionic monoamniotic twins, 3 trichorionic triamniotic triplets, and 2 dichorionic triamniotic triplets. The characteristics of the mothers and their newborns are shown in Table 1 and Table 2. Birth weight, length, and head circumference were registered at the time of birth using standard anthropometric procedures. The length of pregnancy was calculated based on the first day of the last menstrual period according to Naegel’s rule and determined by the crown-rump length (CRL) measured using ultrasonography in the first trimester. The anthropometric and biological characteristics of the mothers (age, weight, morphological blood analysis) and the infants (shoulder width, weight, length, head circumference, length of pregnancy, and sex) and the weight were taken from medical records.

Information about socio-demographic characteristics, cigarette smoking before pregnancy, the use of dietary supplements, and obstetrical and gynecological histories, including parity (number of deliveries) was gathered through general questionnaires. Most women (*n* = 40) took the supplement Prenatal DUO©, consumed once daily, as assessed during medical interviews at periodic meetings. Information on maternal diet during pregnancy was not available. In our study, 73.7% of the women never smoked and 26.3% reported they had stopped smoking either before pregnancy or during the first trimester.

Placentas were collected immediately after delivery, and then weighed and measured. A representative 10–15 cm-long sample was excised from the middle of the radius (distance between the insertion of the umbilical cord and the periphery) without fetal and maternal membranes. Fetal membranes and umbilical cords were taken in their entirety for the study.

### 2.3. Determination of Metals in Afterbirths

The placentas (*n* = 107), umbilical cords (*n* = 114), and fetal membranes (*n* = 112) were collected immediately after delivery, and then weighed and measured, and stored at −30 °C until the study group had been gathered. Before the analysis, the samples were restored to room temperature, dried for three weeks at 55 °C, and later at 105 °C for seven days to a constant weight. The prepared material was ground into a powder in a porcelain mortar and 0.2 g was added to 5 mL of 65% HNO_3_ Suprapur (Merck, Kenilworth, NJ, USA) and 1 mL of non-stabilized 30% H_2_O_2_ solution Suprapur (Merck, Kenilworth, NJ, USA) in clean polypropylene tubes. The reagents were added to 333 vials, and each sample was allowed a 30 min pre-reaction time in a clean room. Once the addition of all the reagents was complete, the 333 samples were placed in Teflon vessels and heated in a microwave digestion system (MARS 5, CEM). The samples were then transferred to acid-washed 15 mL polypropylene tubes. The Ca, K, Na, and Mg levels were determined using inductively coupled plasma atomic emission spectroscopy (ICP-OES) using an ICAP 7400 Duo, Thermo Scientific (Waltham, MA, USA). Analysis was performed in axial mode. Final 25-fold and 5-fold dilutions were performed before ICP-OES measurement. Blank samples were prepared by adding concentrated nitric acid to tubes without a sample and diluting them in the same manner as the test samples. Multi-element calibration standards (ICP multi-element standard solution IV, Merck, Germany) were prepared with different concentrations of inorganic elements in the same manner as the blanks and samples. Deionized water Direct Q UV (Millipore, Burlington, MA, USA) approximately 18.0 MΩ was used to prepare all solutions. The reliability of the analytical procedure was controlled by the determination of elements in reference material with a known concentration: Bovine Muscle NIST-SRM 8414 reference material (National Institute of Standards and Technology, Gaithersburg, MD, USA) and the recovery of internal standard (yttrium; Y) (Table 3).The concentration values of the reference materials given by the manufacturers and our determinations are shown in Table 3. The recovery of Y was 89–105%. The r^2^ values for all standard curves ranged between 0.998 and 1.000. To eliminate possible interference, the emission lines were selected empirically in pilot measurements. The wavelengths (nm) were Ca = 315.887, K = 766.490, Na = 589.592, and Mg = 280.270. The Ca, K, Na, and Mg concentrations in the placenta, the umbilical cord, and the fetal membrane are presented in mg/kg^−1^ dry mass (dw).

### 2.4. Statistical Analysis

Statistical analysis was performed using Statistica v13.0 (Stat Soft). Shapiro–Wilk analysis was performed to test the normal distribution of the data. Spearman’s (rho, ρ) correlation coefficient was used for nonparametric data, and Pearson correlation coefficients for parametric data were used to determine the statistical significance of differences between groups. The significance level was *p* < 0.05.

## 3. Results

The mean water constituents in the placenta, fetal membrane, and umbilical cord were approximately 83%, 85%, and 88%, respectively. The concentrations of Ca, K, Na, and Mg in the placenta are presented in Table 4. In the pairs of newborns, the total concentrations of Ca, K, Na, and Mg in the tissues can be arranged (according to the AM) in the following descending order:

- for Ca, K, Mg: placenta > fetal membrane > umbilical cord;
- for Na: umbilical cord > fetal membrane > placenta.

The correlations between Ca, K, Na, and Mg concentrations in the tissues are shown in Table 5. In the studied tissues from northwestern Poland, we found strong positive correlations between:

- Ca and Mg concentrations in the umbilical cord (r = 0.81, *p* = 0.00) and fetal membrane (r = 0.73, *p* = 0.00);
- K and Mg concentrations in the placenta (r = 0.73, *p* = 0.00);
- Ca and K concentrations in the fetal membrane (r = 0.73, *p* = 0.00).

Negative correlations were found between Na and Ca concentrations in the fetal membrane (r = −0.40, *p* = 0.00) and between Na concentrations in the fetal membrane and Mg concentrations in the placenta (r = −0.16, *p* = 0.02).

Negative correlations were confirmed between the length of pregnancy and: (i) head circumference (ρ = −0.42; *p* = 0.00), (ii) infant weight (ρ = −0.42; *p* = 0.00), (iii) infant length (ρ = −0.49; *p* = 0.00), (iv) shoulder width (ρ = −0.49; *p* = 0.00), and between the infant weight and (i) head circumference (ρ = −0.62; *p* = 0.00), (ii) weight before delivery (ρ = −0.36; *p* = 0.00), (iii) infant length (ρ = −0.45; *p* = 0.00), (iv) shoulder width (ρ = −0.63; *p* = 0.00), and (v) weight gain during pregnancy (ρ = −0.31; *p* = 0.01). We found statistically significant correlations between cigarette smoking before pregnancy and the women’s weight before delivery (ρ = 0.32, *p* = 0.00), and a negative correlation between the women’s ages and infant head circumference (ρ = −0.20, *p* = 0.02) (Table 6).

In the study, we determined correlations between Ca, K, Na, and Mg concentration in the placenta, umbilical cord, and fetal membrane and the parameters of the infants, length of pregnancy, and cigarette smoking before pregnancy (Table 7). We found a moderately positive correlation between:

- placental Ca concentration and placental weight (ρ = 0.42, *p* = 0.00);
- umbilical cord Mg concentrations and the length of the pregnancy (ρ = 0.42, *p* = 0.00).

## 4. Discussion

Intracellular ions (K and Mg) and extracellular ions (Na and Ca) affect pregnancy and child development. The determination of the concentrations of essential elements in the afterbirth allows a retrospective assessment of their effect on pregnancy and has been the subject of research. Most often, the concentration of these elements is determined in maternal blood and umbilical cord blood; there are limited data on the concentration of these elements in the afterbirth tissues.

In the study presented here, a negative correlation was found between head circumference and the mother’s age (Table 6). A similar correlation was found by Di Gravio et al. [35], who showed reduced brain growth in the newborns of younger mothers. Despite the notable differences in fetal head size between younger and older mothers, the authors concluded that this did not affect the children’s health.

### 4.1. The Ca, K, Na, and Mg Concentrations in Afterbirth Tissues

#### 4.1.1. Calcium

In the presented study, the average Ca concentration in the placenta from multiple pregnancies was 1748 mg/kg^−1^ dw. In singleton pregnancies, lower Ca levels were found in women around 30 years of age from Poland (1004 mg/kg^−1^ dw) [24] and Jamaica (846 mg/kg^−1^) [36]. This may indicate that placental Ca concentrations are higher in multiple pregnancies than in singleton pregnancies, confirming the findings by de Angelis et al. [37], where the concentrations of Ca in the placenta of women with single and multiple pregnancies were 92 and 206 mg/kg^−1^ dw, respectively. This could suggest that women with multiple pregnancies are more likely to use dietary supplements and are more concerned with a proper diet. However, Kot et al. [38] found a much higher concentration of Ca in the placenta in women with a singleton pregnancy from the same area (3474 mg/kg^−1^ dw). The concentration of Ca in the placenta may also be influenced by the woman’s age. De Moraes et al. [39] noted that in the placenta of teenage women with singleton pregnancies, Ca concentrations were lower than in adult women, med. at 512 and 2035 mg/kg^−1^, respectively.

In the study presented here, Ca concentrations in the fetal membrane and umbilical cords were 791 and 927 mg/kg^−1^ dw, respectively. In contrast, Kot et al. [38] found much higher Ca levels in the fetal membrane (1665 mg/kg^−1^ dw) and lower Ca levels in the umbilical cord (852 mg/kg^−1^ dw) in singleton pregnancies.

The present study showed an indirect effect of Ca level on weight, shoulder width, head circumference, and baby length (Table 7). In a similar study conducted on women with singleton pregnancies, Grant et al. [36] found a positive correlation between placental Ca levels and newborn weight. Doi et al. [40] showed that Ca concentration in the umbilical cord blood is related to birth length and fetal growth. Khoushabi et al. [41] showed that maternal serum Ca concentration relates to the newborn’s birth weight. The studies by Elizabeth et al. [42] and Bogden et al. [43] confirmed that low birth weight is associated with low Ca concentration in umbilical cord blood. Although hypocalcemia is not a significant problem during pregnancy, the need to develop regimens for managing such patients has been noted [44]. Insufficient Ca concentrations, most often caused by primary hyperparathyroidism (PHPT), can lead to intrauterine growth restriction, the death of the offspring, and severe neonatal hypocalcemia [45]. On the other hand, Liebgotta and Srebrola [46] showed in a laboratory study on rats that continuous access to a Ca-rich diet resulted in reduced fetal weight and delayed skeletal calcification in the fetus.

The present study also found a positive correlation between the umbilical cord Ca concentration and the duration of the pregnancy. Thus, it can be speculated that Ca deficiency may shorten pregnancy time, but no other studies confirm these observations. Malas and Shurideh [47] showed that low Ca levels during pregnancy can result in postpartum hypertension. Ephraim et al. [48] showed that low Ca levels significantly affect the occurrence of pregnancy-induced hypertension and pre-eclampsia. The experimental study by DeSouse et al. [49], on trophoblastic debris taken from the placentas of women with pre-eclampsia, confirmed the effect of Ca in preventing endothelial cell activation induced by trophoblastic debris from pre-eclamptic placentae. In contrast, hypercalcemia, a rare condition and most often occurring during primary hyperparathyroidism (PHPT) [50,51], can also significantly shorten a pregnancy, termination in the course of pre-eclampsia, or neonatal death due to hypoparathyroidism [45,52].

#### 4.1.2. Potassium

In the study presented here, average K concentrations in placentas collected from multiple pregnancies were 8873 mg/kg^−1^, and those in the fetal membrane and umbilical cord were 6173 and 7460 mg/kg^−1^, respectively. Similar results for K concentrations in the placenta were shown by Mazurek et al. [24], ranging from 1138 to 10,093 mg/kg^−1^.

The concentrations of K in the placenta, the umbilical cord, and the fetal membrane significantly correlated with the physical parameters of the infants (Table 7). However, there are not many scientific studies on the direct effects of K on the fetus. In a similar study involving women with singleton pregnancies, Grant et al. [36] found no correlation between placental K concentration and infant weight. In contrast, the authors found a negative correlation between K concentration and neonatal birth weight, which may have been related to low arterial blood pressure. Bell et al. [53] showed that an unregulated supply of K (especially from the environment) could affect an infant’s birth weight and, thus, its morphometric parameters. Similar results were obtained by Mazurek et al. [24], who proved that higher concentrations of K in the mother’s placenta resulted in a low birth weight. In the present study, K concentration was found to significantly affect the pregnancy’s duration, an effect that has been linked to the association of K with severe pre-eclampsia [54]. Low K concentrations, especially in the first half of a pregnancy, are significantly associated with a lower risk of severe pre-eclampsia [55].

#### 4.1.3. Sodium

In the present study, average Na concentrations in the placenta in multiple pregnancies were 9323 mg/kg^−1^ dw, and in the fetal membrane and umbilical cord, 26,757 and 26,616 mg/kg^−1^ dw, respectively.

Sodium affects fetal growth, increases cell mass, and stimulates cell proliferation and protein synthesis. The direct effect of Na deficiency on child development is confirmed by studies in pregnant women [56] and laboratory animals [57,58]. In the study presented here, we found that the Na concentration in the umbilical cord significantly influenced fetal weight, shoulder width, body length, and the head circumference of the baby (Table 7). Similarly, the placental Na concentration greatly affected the weight, body length, shoulder width, and length of the baby (Table 7). Likewise, Lagiou et al. [59] found that the pregnant woman’s optimal Na intake significantly affected the baby’s morphometric parameters, especially the baby’s birth weight. In contrast, Grant et al. [36], studying women with singleton pregnancies, found no relationship between placental Na concentration and the newborn’s weight.

Na concentration in the umbilical cord showed a positive linear correlation with the duration of the pregnancy. A low level of Na (hyponatremia) is the most common electrolyte abnormality that may pose a risk to pregnancy [60] and contribute to pre-eclampsia, which can result in a shorter pregnancy [61,62,63]. Hsu et al. [64] showed that hyponatremia in pre-eclampsia can lead to maternal respiratory and circulatory arrest. This is also confirmed by Powel et al. [65], based on a case report of a twin pregnancy and data from the literature showing that pregnant women should be screened for water–electrolyte balance as part of a risk assessment for pre-eclampsia, as elevated Na levels can also be a risk factor for this condition [66].

#### 4.1.4. Magnesium

In the study presented here, the average concentration of Mg in the placenta taken from women with multiple pregnancies was 436 mg/kg^−1^ dw. In singleton pregnancies, a much lower concentration of Mg was found by Mazurek et al. [24] in placentas, ranging from 66 to 377 mg/kg^−1^. In comparison, in a study conducted in the US, de Angelis et al. [37] found that the concentration of Mg in placentas from multiple pregnancies (56 mg/kg^−1^) was higher than for singleton pregnancies (23 mg/kg^−1^). In the study by Osada et al. [67], conducted on a group of singleton pregnancies with an appropriate gestational age (AGA), the Mg concentration was 18 µmol/l, while in a group of singleton pregnancies with intrauterine growth retardation (IGUR), the Mg concentration was 21 µmo/l µmol/L. In our study, Mg concentrations in the fetal membrane and umbilical cords were 326 and 324 mg/kg^−1^, respectively.

In the present study, we found that Mg concentrations in the fetal membrane and umbilical cord significantly correlated with the baby’s weight, length, shoulder width, and body length centile (Table 7). Hypomagnesemia in pregnancy is quite common, especially in developing countries [68], which significantly increases the chances of low birth weight [69] and intrauterine fetal growth retardation (IUGR) [70,71,72]. Laboratory studies in mice have confirmed the adverse effects of Mg deficiency on placental and fetal development [73,74]. Kazemi-Darabadi and Akbari [75] confirmed in a rat model a difference between an Mg-supplemented animal group and a control group in morphometric parameters, such as weight, length, and the shoulder width of the fetuses. Enaruna et al. [68] studied pregnancies in a university hospital in Benin and showed that hypomagnesemia significantly contributes to pre-eclampsia and preterm labor. In the present study, the results indirectly showed that Mg substantially influenced the length of a pregnancy. Preterm labor is associated with uterine muscle cell hyper-reactivity [26,76] or endothelial dysfunction [77]. Kovo et al. [78] confirmed the significant anti-inflammatory effect of Mg in an experimental study on placentas, and selected cotyledons were cannulated. Additionally, they showed that Mg administered in the form of magnesium sulfate (MgSO_4_) blocked an increase in the levels of proteins involved in the inflammatory cascade, including the nuclear factor K light chain enhancer of the activated B cells (NF-κB), interleukin (IL) 6, adrenocorticotropic hormone (ACTH), and nitric oxide synthase (NOS).

### 4.2. The Influence of Tobacco Smoke

The mother’s lifestyle during pregnancy has a significant impact on her own and her child’s health [79]. Smoking cigarettes during pregnancy has been found to shorten the gestation period and cause lower birth weight and body length [80,81,82]. The most serious consequences of smoking during pregnancy are intrauterine fetal death and an increased risk of sudden infant death syndrome (SIDS) in the first months of life [83]. In the present study, none of the women smoked cigarettes during pregnancy, but 15 women had smoked cigarettes before pregnancy. The present study found an association between smoking cigarettes before pregnancy and women’s higher weight just before delivery. Hulman et al. [84] and Adegboye et al. [85] noted that quitting smoking during pregnancy results in significant weight gain for pregnant women. Unfortunately, none of these studies apply to women who ceased smoking before pregnancy. Smoking women had higher Ca, P, K, Mg, Fe, Cu, and Cd concentrations in the placental tissue than non-smokers [24].

### 4.3. Limitations of This Study

The limitations of this study are associated with its design, which was intended to present general trends and provide hypotheses for further research in this field. It evaluated Ca, K, Na, and Mg concentrations in the placenta, umbilical cord, and fetal membrane, to obtain information on the influence of the elements on the morphometric parameters of the baby and the duration of the pregnancy, as well as mutual correlations showing their antagonistic/non-antagonistic effects. However, it lacks the determination of the parameters of the renin-angiotensin-aldosterone system responsible for controlling potassium–sodium balance.

This paper did not analyze the parameters responsible for Ca absorption, such as vitamin D3 1,25- dihydroxycholecalciferol (1,25-(OH)_2_D_3_, calcitriol), parathyroid hormone (PTH), and calcitonin. In addition, the data on dietary supplements and smoking were collected through a voluntary questionnaire. Diet was not strictly controlled. Further limitations include the lack of assessment of the uterine position, vascularization, and function by Doppler and biochemical tests, i.e., PLGF.

Twin data provide a unique opportunity to analyze data related to pregnancies. However, using appropriate statistical methods is fundamental for exploiting such potential. However, in this study, we mainly wanted to show the correlation between the concentration of calcium, potassium, sodium, and magnesium in the placenta, umbilical cord, and fetal membrane of women with multiple pregnancies. The main limitations of using selected “simple” statistical methodology are that it requires a binary exposure, adaptations for binary outcomes and sub-optimal inference, confounders cannot be included, and it is unsuitable for repeated measurements. Moreover, samples were treated as independent.

The primary intention was to illustrate approaches for further research on micronutrients in multiple pregnancies, which are just beginning to be studied due to the annual increase in the percentage of multifetal pregnancies compared to singleton pregnancies [86]. Our research may indicate the way for further studies, but it should be taken into consideration that all those elements are under homeostatic control in body fluids and are influenced by underlying (patho)physiological factors of the organism as well as personal nutritional and lifestyle habits

## 5. Conclusions

The conclusions of this study should be interpreted conservatively/carefully, due to the many limitations of the study and the simple statistics. However, it adds to the knowledge of elemental concentrations in multiple pregnancies and their possible effects on fetal morphometric parameters.

## Figures and Tables

**Table 1:** Maternal and neonatal characteristics (AM, arithmetic mean; SD, standard deviation; Med, median; *n*, number of participants).

Parameter		AM ± SD	Med	Range
maternal characteristics:				
age (years)		31.0 ± 4.8	31	21–41
weight (kg) before pregnancy		66.7 ± 16.4	63	45–134
weight (kg) before delivery		84.4 ± 18.3	82	58–140
weight gain during pregnancy		18.7 ± 8.6	16.0	8–38
neonatal characteristics:				
length of pregnancy (weeks)		34.6 ± 2.5	35	26–38
birth weight (g)		2247 ± 497	2330	690–3350
length (cm)		47.8 ± 4.3	48	29–55
head circumference (cm)		31.9 ± 2.2	32	24–37
shoulder width (cm)		29.1 ± 3.0	29	18–36
placenta weight (g)	dichorionic diamniotic twins pregnancy (*n* = 40)	1102 ± 293	1200	510–1300
	monochorionic diamniotic twins pregnancy (*n* = 11)	1160 ± 230	1200	800–1400
	monochorionic monoamniotic twins pregnancy (*n* = 1)	420	420	420
	dichorionic triamniotic triplets pregnancy (*n* = 3)	700 ± 57.7	700	650–750
	trichorionic triamniotic triplets pregnancy (*n* = 2)	571 ± 212	642	280–780

**Table 2:** Smoothed centiles for birth weight and birth length of the boys (*n* = 54) and girls (*n* = 65) (Fenton Growth Chart).

Centiles for Length (cm)	Boys	Girls	Total	Centiles for Birth Weight (kg)	Boys	Girls	Total
>3 or <97	40	50	90	>3 or <97	51	59	110
<3 or >97	14	15	29	<3 or >97	3	6	9

**Table 3:** Analysis of reference material Bovine Muscle NIST-SRM 8414.

Element	Reference Values (mg/L)	Percentage of Reference Values
Ca	145 ± 20	95.9
K	15,170 ± 370	100.9
Na	2100 ± 80	103.5
Mg	960 ± 95	102.7

**Table 4:** Concentrations of sodium (Na), magnesium (Mg), calcium (Ca), and potassium (K) in the placenta, umbilical cord, and fetal membrane (AM, arithmetic mean; Med., median; Max, maximum; Min, minimum; SD, standard deviation) (in mg/kg^−1^ dry mass, dw).

	Placenta			Umbilical Cord			Fetal Membrane		
	AM ± SD	Med.	Range	AM ± SD	Med.	Range	AM ± SD	Med.	Range
Twins (*n* = 52)									
Ca	2498 ± 2020	1659	531–8802	937 ± 223	913	539–1571	1206 ± 1088	764	403–7448
K	8895 ± 2038	8991	3928–14,466	6147 ± 1530	6015	3195–11,094	7440 ± 1999	7427	2959–12,713
Na	9272 ± 1915	8899	4493–16,519	25,850 ± 6949	25,267	10,773–48,850	13,385 ± 3484	13,207	7913–21,879
Mg	436 ± 110	421	229–808	322 ± 62.0	321	215–560	373 ± 112	362	180–839
Triplets (*n* = 5)									
Ca	2269 ± 1794	2004	823–7753	1140 ± 219	1192	774–1463	1544 ± 955	1313	486–3367
K	8736 ± 1871	9317	5969–12,329	6410 ± 1440	5875	4874–9900	7589 ± 2111	7685	3511–11,177
Na	9647 ± 1528	7267	7267–12,520	35,085 ± 8155	31,735	26,606–48,539	14,711 ± 4259	13,893	9835–22,296
Mg	438 ± 79	473	326–530	369 ± 48.3	378	288–430	354 ± 61.2	349	278–450
Total (*n* = 57)									
Ca	2466 ± 1983	1748	530–8802	957 ± 230	927	539–1571	1 252 ± 1073	791	403–7448
K	8873 ± 2009	8992	3928–14,466	6173 ± 1517	5985	3195–11,094	7460 ± 2005	7655	2959–12,713
Na	9323 ± 1865	9007	4493–16,519	26,757 ± 7558	26,616	10,773–48,850	13,562 ± 3602	13,231	7913–22,296
Mg	436 ± 106	423	229–808	326 ± 62.2	324	215–560	370 ± 107	362	180–839

**Table 5:** Pearson’s coefficients for correlations between Ca, K, Na, and Mg concentrations in the placenta (P), umbilical cord (UC), and fetal membrane (FM).

	Ca_UC_ (p)	Ca_FM_ (p)	K_P_ (p)	K_UC_ (p)	K_FM_ (p)	Mg_P_ (p)	Mg_UC_ (p)	Mg_FM_ (p)
**Ca_FM_**		x	0.47 (0.00)		0.73 (0.00)	0.27 (0.01)		0.73 (0.00)
**Ca_P_**			0.20 (0.00)			0.78 (0.00)		
**K_UC_**			0.67 (0.00)	x	0.32 (0.00)	0.33 (0.00)	0.39 (0.00)	0.45 (0.00)
**K_FM_**		0.73 (0.00)		0.32 (0.00)	x	0.62 (0.00)		0.45 (0.00)
**Na_P_**			0.36 (0.00)	0.34 (0.00)	0.39 (0.00)	0.50 (0.00)	0.21 (0.04)	0.48 (0.00)
**Na_UC_**	0.34 (0.00)			0.22 (0.02)			0.52 (0.00)	
**Na_FM_**		−0.40 (0.00)				−0.16 (0.02)		
**Mg_P_**		0.27 (0.01)	0.73 (0.00)	0.33 (0.00)	0.62 (0.00)	x		
**Mg_UC_**	0.81 (0.00)		0.27 (0.00)	0.39 (0.00)			x	0.23 (0.02)
**Mg_FM_**		0.73 (0.00)	0.55 (0.00)	0.45 (0.00)	0.45 (0.00)		0.23 (0.02)	x

**Table 6:** Spearman’s coefficients for correlations between anthropometric parameters of the infants, intake of supplements, and selected characteristics of the mothers.

	Head Circumference (p)	Infant Weight (p)	Infant Length (p)	Shoulder Width (p)	Centiles for Length (p)	Length of Pregnancy (p)	Placenta Weight (p)	Weight before Delivery (p)	Weight Gain during Pregnancy (p)	Weight before Pregnancy (p)
**Neonatal characteristics**										
centiles for birth weight	−0.34 (0.00)	0.21 (0.02)	−0.34 (0.00)	−0.35 (0.00)	0.28 (0.00)			−0.19 (0.04)	−0.25 (0.04)	
head circumference	x	−0.62 (0.00)	0.57 (0.00)	0.76 (0.00)		−0.42 (0.00)	0.18 (0.05)	0.21 (0.02)	0.31 (0.01)	
infant weight	−0.62 (0.00)	x	−0.45 (0.00)	−0.63 (0.00)		−0.42 (0.00)		−0.36 (0.00)	−0.31 (0.01)	
infant length	0.57 (0.00)	−0.45 (0.00)	x	0.66 (0.00)		−0.49 (0.00)		0.20 (0.03)	0.28 (0.02)	
shoulder width	0.76 (0.00)	−0.63 (0.00)	0.66 (0.00)	x		−0.49 (0.00)	0.23 (0.01)	0.25 (0.01)		
Maternal characteristics										
weight before delivery	0.21 (0.02)	−0.36 (0.00)	0.20 (0.03)	0.25 (0.01)			0.22 (0.02)	x	0.64 (0.00)	0.81 (0.00)
cigarette smoking before pregnancy								0.32 (0.00)		
age of the women	−0.20 (0.02)									

**Table 7:** Spearman’s coefficients for correlations between the Ca, K, Na, and Mg concentrations in the placenta (P), umbilical cord (UC), and fetal membrane (FM) and anthropometric parameters of the infants, length of pregnancy, placenta weight, and cigarette smoking.

	Ca_P_ (p)	Ca_UC_ (p)	Ca_FM_ (p)	K_P_ (p)	K_UC_ (p)	K_FM_ (p)	Na_P_ (p)	Na_UC_ (p)	Mg_FM_ (p)	Mg_UC_ (p)
length of pregnancy		0.34 (0.00)		0.34 (0.00)	0.24 (0.01)	0.22 (0.02)		0.25 (0.01)	0.20 (0.04)	0.42 (0.00)
infant weight					0.20 (0.03)			0.20 (0.03)		0.26 (0.01)
shoulder width		−0.23 (0.01)	−0.21 (0.03)	−0.26 (0.01)	−0.24 (0.01)	−0.23 (0.02)	−0.29 (0.01)	−0.25 (0.01)	−0.25 (0.01)	−0.29 (0.01)
infant length		−0.24 (0.01)		−0.20 (0.04)		−0.24 (0.01)	−0.22 (0.02)	−0.21 (0.03)	−0.20 (0.04)	−0.27 (0.01)
centiles for length							0.24 (0.01)			0.22 (0.02)
placenta weight	0.42 (0.00)									
head circumference								−0.22 (0.02)		
cigarette smoking before pregnancy			−0.24 (0.01)					−0.24 (0.01)		

## Data Availability

The data presented in this study are available on request from the corresponding author.
